# Enzymatic characterization of three human RNA adenosine methyltransferases reveals diverse substrate affinities and reaction optima

**DOI:** 10.1016/j.jbc.2021.100270

**Published:** 2021-01-09

**Authors:** Dan Yu, Gundeep Kaur, Robert M. Blumenthal, Xing Zhang, Xiaodong Cheng

**Affiliations:** 1 Department of Epigenetics and Molecular Carcinogenesis, University of Texas MD Anderson Cancer Center, Houston, Texas, USA; 2 Department of Medical Microbiology and Immunology, and Program in Bioinformatics, The University of Toledo College of Medicine and Life Sciences, Toledo, Ohio, USA

**Keywords:** RNA adenine methylation, PCIF1, MettL5-Trm112, MettL16, DLS, dynamic light scattering, GST, glutathione S-transferase, ITC, isothermal titration calorimetry, MTase, methyltransferase, PDB, Protein Data Bank, SAH, S-adenosyl-L-homocysteine, SAM, S-adenosyl-L-methionine, SAXS, small-angle X-ray scattering

## Abstract

RNA methylations of varied RNA species (mRNA, tRNA, rRNA, non-coding RNA) generate a range of modified nucleotides, including N6-methyladenosine. Here we study the enzymology of three human RNA methyltransferases that methylate the adenosine amino group in diverse contexts, when it is: the first transcribed nucleotide after the mRNA cap (PCIF1), at position 1832 of 18S rRNA (MettL5-Trm112 complex), and within a hairpin in the 3′ UTR of the S-adenosyl-l-methionine synthetase (MettL16). Among these three enzymes, the catalytic efficiency ranges from PCIF1, with the fastest turnover rate of >230 h^−1^ μM^−1^ on mRNA cap analog, down to MettL16, which has the lowest rate of ∼3 h^−1^ μM^−1^ acting on an RNA hairpin. Both PCIF1 and MettL5 have a binding affinity (*K*_m_) of ∼1 μM or less for both substrates of SAM and RNA, whereas MettL16 has significantly lower binding affinities for both (*K*_m_ >0.4 mM for SAM and ∼10 μM for RNA). The three enzymes are active over a wide pH range (∼5.4–9.4) and have different preferences for ionic strength. Sodium chloride at 200 mM markedly diminished methylation activity of MettL5-Trm112 complex, whereas MettL16 had higher activity in the range of 200 to 500 mM NaCl. Zinc ion inhibited activities of all three enzymes. Together, these results illustrate the diversity of RNA adenosine methyltransferases in their enzymatic mechanisms and substrate specificities and underline the need for assay optimization in their study.

Postsynthetic methylations of DNA and RNA are common and are well known to play significant roles in a wide range of cellular functions in bacterial and archaea (1, 2, 3), such as adenine methylation-directed mismatch repair in *Escherichia coli* (4). These enzyme-driven chemical reactions use S-adenosyl-l-methionine (SAM) as the methyl donor and transfer the methyl group onto DNA or RNA at the ring carbon C5 of cytosine (yielding 5-methylcytosine, 5mC) or at the exocyclic amino groups of either cytosine at N4 (yielding N4-methylcytosine, N4mC) or adenine at N6 (yielding N6-methyladenine, N6mA) (5). [There are also methyltransferases (MTases) that modify other parts of the nucleic acid (*e.g.*, (6) and references therein), but here we are considering only modification of the bases.]

Mammalian DNA 5mC is a major epigenetic regulator in development and disease (7), while in mammals the amino modification of cytosine in DNA (N4mC) has not been established and that of adenine in DNA (N6mA) is controversial. While noted immunochemically as early as 1983 (8), N6mA was reported in mammalian DNA using very sensitive approaches only in 2016 (9), and its existence in mammals is still debatable (10, 11). Remaining unsettled questions include how N6mA is generated in mammalian DNA (12, 13) and identification of the potential adenine DNA MTase(s) (14, 15, 16).

In contrast, the RNA methylation generating N6mA has been found in most eukaryotic RNA molecules including mRNA, tRNA, rRNA, noncoding RNA, and chromosome-associated regulatory RNA (17, 18, 19, 20, 21). Here, we chose three recently identified human RNA adenine MTases: PCIF1, acting on mRNA, MettL5 on rRNA, and MettL16 on snRNA. PCIF1—named as phosphorylated RNA polymerase II CTD interacting factor 1 (22)—methylates adenosine when it is the first transcribed nucleotide after the mRNA cap (23, 24, 25, 26). PCIF1 affects mRNA levels in mouse (27), though this effect might be mediated *via* its direct interaction with the phosphorylated C-terminal domain of RNA polymerase II, as has been demonstrated for the mouse, human, and *Drosophila* orthologs (27, 28). MettL5 forms a heterodimer with Trm112, a conserved protein that binds to and stabilizes various MTase proteins and was named initially for its role in tRNA methylation (29, 30). The MettL5-Trm112 complex is responsible for 18S rRNA adenine methylation at position 1832 (31, 32, 33, 34, 35). Finally, MettL16 catalyzes adenine methylation in the conserved sequence UAC**A**GAGAA, within hairpins in the 3′ UTR of the SAM synthetase (MAT2A) mRNA and in U6 snRNA (36, 37, 38). The discovery of adenine methylation is often aided by the sensitive mass spectrometry approach (23, 26, 32, 34, 36). We note that these three MTases all possess conserved sequence motifs for binding cofactor SAM (motif I) and for catalysis of amino (NH_2_)-methylation (motif IV) in a particular order in their amino acid sequence (39) and retain the characteristic overall fold of seven-stranded class-I MTases (40) (Fig. S1). Here, we found a surprisingly wide range of optimal conditions for the *in vitro* enzymatic activity of each enzyme acting on its known RNA substrates.

## Results

### PCIF1 has a unique optimum pH on mRNA cap analog

**Figure 1:**
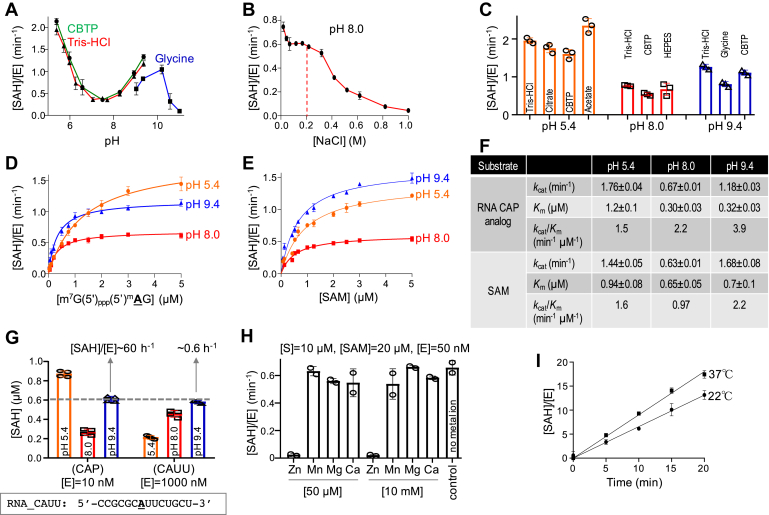
**Kinetics of PCIF1 on mRNA cap analog.***A*, PCIF1 activity as a function of pH, presented as the formation of byproduct SAH concentration per enzyme molecule [SAH]/[E], in a bioluminescence assay. The citrate Bis-Tris propane buffer (*green line*), used for pH values below 9.4, contained a mixture of 10 mM citric acid and 10 mM BisTris propane. Two additional buffers were used: 20 mM Tris-HCl for pH values below 9.4 (*red line*), and 20 mM glycine for pH values above 9.0 (*blue line*). *B*, PCIF1 activity as a function of NaCl concentration at pH 8.0. *C*, PCIF1 activities at three pH conditions using different buffering agents. *D* and *E*, comparison of PCIF1 activities at three pH conditions by varying concentrations of substrate RNA (*D*) or SAM (*E*). The dependence of the velocity of product SAH formation, per enzyme molecule, on substrate concentration was analyzed according to the Michaelis–Menten equation. *F*, summary of PCIF1 kinetic parameters. Data represent the mean ± SD of two independent determinations, with duplicates assayed for each of the two determinations. *G*, comparison of PCIF1 activities on mRNA cap analog and an RNA oligo containing a single A. *H*, Inhibitory effect of zinc on PCIF1 activity on cap RNA analog. *I*, comparison of PCIF1 activity on cap RNA analog at 37 °C and room temperature (∼22 °C).

We first optimized the enzymatic activity of purified recombinant full-length human PCIF1 (Fig. S2, *A*–*C*), using as substrate the O-methylated cap analog, which has a 2’-O-methyladenosine at the +1 site [m7G(5’)_ppp_(5’)(2’O_me_**A**)_p_G]. PCIF1 is active over a wide pH range of ∼5 to 10 (Fig. 1*A*), but is sensitive to increased ionic strength of sodium chloride beyond 200 mM (Fig. 1*B*). Interestingly, we observed greater activity at both ends of the tested pH range, at either pH 5.4 or pH 9.4 (Fig. 1*A*), which was independent of the buffering agent used (Fig. 1*C*).

We next measured the PCIF1 kinetic parameters at three different pH values (5.4, 8.0, and 9.4) by varying, respectively, concentrations of the RNA substrate (Fig. 1*D*) and methyl donor SAM (Fig. 1*E*). Like the pH activity curve, the *k*_cat_ values for the RNA and SAM substrates are each ∼2 to 3× higher at pH 5.4 and 9.4 than at pH 8.0 (summarized in Fig. 1*F*). However, binding affinities for the RNA substrate (as reflected by *K*_m_ values) are approximately the same (∼0.3 μM) at the two higher pH conditions (8.0 and 9.6) and fourfold lower (*i.e.*, increased binding affinity) than at pH 5.4 (*K*_m_ value ∼1.2 μM). For comparison, Akichika *et al.* (23) reported *K*_m_ = 3.5 μM for m7G_ppp_(2’O_me_**A**). That *K*_m_ for the cap substrate is about an order of magnitude higher than the one we determined (0.3 μM at pH 8.0 *versus* 3.5 μM at pH 7.5). Their study was performed in a reaction mixture containing 50 mM HEPES-KOH [pH 7.5, so near where we saw lowest activity (Fig. 1*A*)].

On the other hand, the binding affinities for SAM (again, as reflected by *K*_m_) remain relatively constant at ∼0.7 to 0.9 μM over a range of pH values. Taken together, the catalytic efficiency of PCIF1 on the mRNA cap analog (comparing *k*_cat_/*K*_m_ values) is the highest at pH 9.4 (3.9 min^−1^ μM^−1^; Fig. 1*F*), more than 1.5× higher than that at pH 8.0 (2.2 min^−1^ μM^−1^) and >2.5× higher than at pH 5.4 (1.5 min^−1^ μM^−1^).

The higher *k*_cat_ value at lower pH (5.4) is probably unique to the cap analog, as there are two titratable groups on the ligand (41) that might be directly involved in the binding. Direct interactions with the cap have not been characterized structurally with human PCIF1, but the zebrafish ortholog uses both positively and negatively charged residues (Arg^269^ and Glu^563^) in its interactions with the ribose and guanine moieties of m7G, as well as Arg^239^ interaction with one of the phosphate groups in the cap (23). These charged residues are all conserved in human PCIF1. We did not observe a similar *k*_*cat*_ *versus* pH phenomenon on a short RNA oligonucleotide—its methylation increased monotonically with increasing pH (Fig. 1*G*). Compared with the mRNA cap analog, we observed greatly reduced activity (by a factor of ∼100) on the conventional RNA oligo (without any pre-modifications) (Fig. 1*G*).

### Biophysical characterization of PCIF1

**Figure 2:**
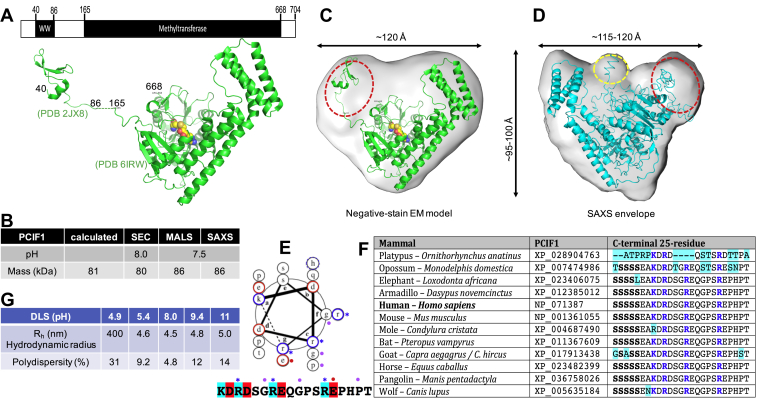
**PCIF1 is globular in solution.***A*, schematic of human PCIF1 with a smaller N-terminal WW domain and a larger C-terminal methyltransferase domain that includes a bound SAH molecule (*yellow*). *B*, summary of mass measurements by size-exclusion chromatography and two biophysical methods (Figs. S2 and S3). *C* and *D*, comparison of low-resolution models generated from negative-stain electron microscopy (panel *C*) and SAXS (panel *D*). The flexible N-terminal residues and linker region between the two domains are indicated by the *dashed red circle*. *C*, the negative-stain EM model with docked X-ray structures of WW domain (PDB 2JX8) and MTase domain in complex with SAH (PDB 6IRW). *D*, the SAXS envelope docked with a homology model of full-length PCIF1 (*cyan*). Note that the C-terminal residues occupy the central bulge of density, indicated by the *dashed yellow circle*. *E*, helical wheel analysis (https://grigoryanlab.org/drawcoil/) of the C-terminal helix. Arginine (r) residues marked with asterisks may form a cationic RNA-binding surface. *F*, the C terminal 25-residues are highly conserved among the Mammalia other than monotremes. Highlighting indicates differences from the human sequence. *G*, summary of dynamic light scattering (DLS) measurements at various pH (N = 3).

Human PCIF1 has been structurally characterized as two separate fragments (23): an NMR structure of the RNA polymerase-binding WW domain (residues 40–86) and a crystal structure of a large C-terminal fragment (residues 165–668) containing the MTase domain in complex with S-adenosyl-l-homocysteine (SAH) (Fig. 2*A*). To exclude the possibility of protein aggregation influencing activity at different pH values, we used three biophysical methods to measure the molecular mass of the full-length PCIF1 at pH 7.5 or 8.0, where we observed the lowest *k*_cat_ value on cap analog (Fig. 1*A*). The sequence-predicted monomeric MW of PCIF1 (704 residues) is 80.7 kDa. First, the samples were subjected to size-exclusion chromatography (SEC), which gave the apparent molecular weight of ∼80 kDa (Fig. S2*A*). Second, SEC coupled with synchrotron-based multiangle light scattering (MALS) (42) gave the absolute mass of 86 kDa with an averaged hydrodynamic radius of 4.8 ± 0.4 nm (Fig. 2*B* and Fig. S2, *E* and *F*). The same SEC fractions were simultaneously examined by synchrotron-based small-angle X-ray scattering (SAXS) (43), which gave the molecular weight of 86 kDa (Fig. 2*B* and Fig. S3). In sum, the observed molecular mass from all three methods agrees with the calculated mass of a monomeric form of PCIF1 (Fig. 2*B*).

The overall shape of the SAXS envelope agrees with a negative stain electron microscopy (EM) model, with the maximum dimension of the molecule to be 115 to 120 Å (Fig. 2, *C* and *D* and Fig. S4). The overall contour of the low-resolution models can be fitted with the X-ray structure of the C-terminal MTase domain (Fig. 2*C*). The additional unaccounted-for density near one corner of the SAXS envelope might be where the N-terminal WW domain is located (red circle in Fig. 2, *C* and *D*). In addition, we observed extra density in the middle of the SAXS envelope, where the nucleic acid substrate might be bound. We generated a homology model for the full-length PCIF1 using I-TASSER (44), including the missing N-terminal residues, the linker region between the WW and MTase domains, and the C-terminal residues. Interestingly, the extreme C-terminal 15-residues form an alpha helix that extends to the central bulged density (Fig. 2*D*). Helical wheel analysis (Fig. 2*E*) suggests that this helix has a basic face that incorporates the three arginine residues in the sequence KD**R**DSG**R**EQGPS**R**EPHPT_COOH_, consistent with possible nucleic acid binding. The C-terminal 25 residues, specifically including these three arginines, are highly conserved among the Mammalia (other than monotremes, Fig. 2*F*). These residues follow a conserved polyserine tract that, in other proteins, can be phosphorylated and affect subnuclear localization (45, 46).

Finally, we used dynamic light scattering to determine the size distribution of PCIF1 at five different pH values (Fig. 2*G*). We observed protein aggregation only at pH < 5, whereas PCIF1 is stable over the pH range of 5.4 to 11, with a hydrodynamic radius of 4.5 to 5 nm (or diameter of ∼9–10 nm) in agreement with SAXS and the EM model (Fig. 2, *C* and *D*). In addition, the protein has the least dynamic nonuniformity, as indicated by smallest polydispersity of ∼5%, at pH 8.0. This suggests that the pH-dependent lower *k*_cat_ value on cap analog is substrate specific.

### MettL5 is active as an RNA adenine methyltransferase *in vitro*

**Figure 3:**
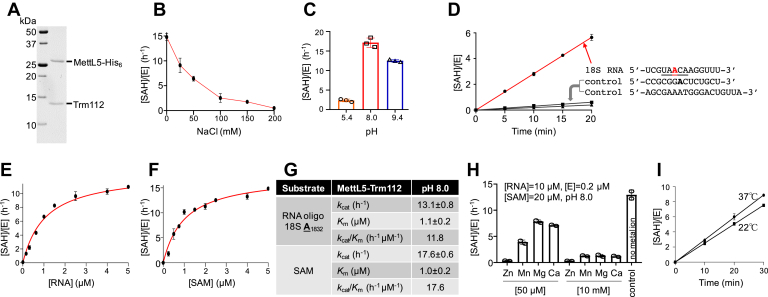
**Kinetics of MettL5-Trm112 on an RNA oligonucleotide representing A**_**1832**_**of 18S rRNA and its flanking nucleotides.***A*, example of purified recombinant MettL5-Trm112 used in the study (18% SDS PAGE with Coomassie blue staining). *B*, activity of MettL5-Trm112, presented as the formation of by-product SAH concentration per enzyme molecule [SAH]/[E] in a bioluminescence assay, is sensitive to NaCl concentration. *C*, MettL5-Trm112 is more active at higher pH conditions. *D*, reaction time 5 to 20 min is within a linear range. Inserted are the 18S RNA sequence and two control RNA oligos used in the study. *E* and *F*, activities of MettL5-Trm112 by varying concentrations of substrate RNA (panel *E*) or SAM (panel *F*). The dependence of the velocity of product SAH formation, per enzyme molecule, on substrate concentration was analyzed according to the Michaelis–Menten equation. *G*, summary of kinetic parameter of MettL5-Trm112. Data represent the mean ± SD of two independent determinations, with duplicates assayed for each of the two determinations. *H*, inhibitory effects of metal ions on MettL5-Trm112 activity. *I*, comparison of MettL5-Trm112 activity on rRNA at 37 °C and room temperature (∼22 °C).

During our study, two publications reported the identification of MettL5, an RNA adenine MTase that methylates A_1832_ of 18S rRNA (31, 32). This 18S rRNA N6mA, first identified about 35 years ago (47), is located in the 3′ minor domain of 18S, at the very base of helix h44, and only a few nucleotides away from the decoding center. Van Tran *et al.* (31) showed that MettL5 must form a heterodimeric complex with Trm112 for its stability in cells and determined an X-ray structure of the MettL5-Trm112 complex with bound SAM (PDB 6H2U). Unexpectedly, despite extensive efforts, the authors “were unable to recapitulate METTL5–TRMT112 enzymatic activity *in vitro* with short single- or double-stranded RNAs corresponding to the sequences surrounding m6A_1832_ in the mature ribosome” (31). On the other hand, a GST-MettL5 fusion purified from *E. coli* (in the absence of Trm112) was active *in vitro* on total RNA, on RNAs >200 nt long, and on dsRNAs (32). To resolve this discrepancy, regarding MettL5 activity *in vitro*, we purified recombinant MettL5-Trm112 complex (Fig. 3*A*).

Because we do not know the conditions under which the unsuccessful experiments were conducted, we designed a short linear 14-mer RNA oligo corresponding to the sequence surrounding A_1832_ of 18S rRNA and tested for *in vitro* MettL5-Trim112 activity under minimal buffer conditions (20 mM Tris-HCl pH 8.0 and 1 mM DTT). We observed activity at no or low NaCl concentration (0–50 mM), but the activity was undetectable at 200 mM NaCl (Fig. 3*B*). Unlike PCIF1, MettL5-Trm112 methylase complex exhibited the highest activity at pH 8.0, followed by pH 9.4 and 5.4 (Fig. 3*C*). Under the linear reaction conditions of pH 8.0 and 50 mM NaCl for 20 min (Fig. 3*D*), the enzyme complex has similar *K*_m_ values (∼1 μM) for the RNA oligo substrate and for SAM, while showing *k*_cat_ values of ∼13 h^−1^ for RNA and 18 h^−1^ for SAM, resulting in catalytic efficiency of ∼12 to 18 h^−1^ μM^−1^ (Fig. 3, *E*–*G*).

### MettL16 activity is inhibited by zinc ion

MettL16, the third N6mA enzyme that we characterized, mediates N6mA methylation in a specific sequence motif (UAC**A**GAGAA) within the terminal loop of a conserved stem-loop structure in the MAT2A 3’ UTR (37). MAT2A encodes isozyme II of the SAM synthetase. MettL16 is a unique MTase in that its binding to the mRNA substrates actually increases under SAM-limiting conditions. This increased binding stability is due to inefficient enzymatic turnover, leading to increased occupancy of the mRNA by the MettL16 protein, which appears to promote MAT2A mRNA splicing. Thus, the unusual sensitivity of MettL16 to SAM levels results in a negative feedback loop, such that increasing SAM levels lead to reduced expression of the SAM synthetase (37). Structurally, MettL16 has been characterized in two binary forms: in complex with the MTA2A RNA hairpin (48) and in complex with SAH (49). Coupled with structural and functional analyses, the *in vitro* enzymatic activity has been assayed in the presence of either ZnCl_2_ (48) or MgCl_2_ (37, 49).

**Figure 4:**
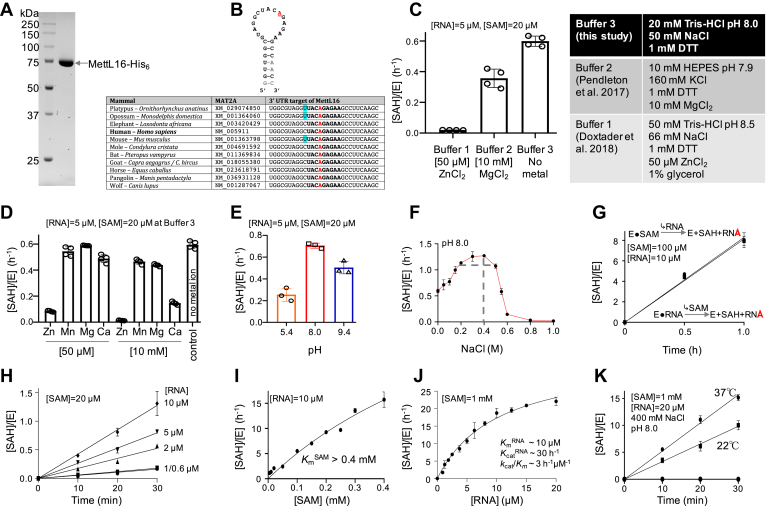
**Activity of MettL16 on MTA2A 3’ UTR RNA hairpin.***A*, example of purified recombinant MettL16 used in the study (12% SDS PAGE with Coomassie blue staining). *B*, the first hairpin of the conserved 3’ UTR of MAT2A mRNA sequences. Shown are the synthetic substrate used (methylated A in *red*) and the orthologous sequences from other Mammalia. Highlighting indicates difference from the human sequence. *C*, MettL16 activities, presented as the formation of byproduct SAH concentration per enzyme molecule [SAH]/[E] in a bioluminescence assay, under three different buffer conditions. *D*, effect of divalent metal ions on MettL16 activity under buffer 3 condition. *E* and *F*, MettL16 has a broad pH range (panel *E*) under 50 mM NaCl and higher activity between 200 and 500 mM NaCl (panel *F*) under pH 8. *G*, MettL16 uses a random sequential reaction, in terms of the order of binding two substrates. *H*, reaction time 10 to 30 min is within a linear range for varied RNA concentrations. *I*, MettL16 has a *K*_m_ value of >0.4 mM for SAM, which is very large for MTases. *J*, MettL16 has a *K*_m_ value of ∼10 μM for RNA. The dependence of the velocity of product SAH formation, per enzyme molecule, on substrate concentration was analyzed according to the Michaelis–Menten equation. *K*, comparison of MettL16 activity on RNA hairpin at 37 °C and room temperature (∼22 °C).

To investigate the metal dependence of MettL16 methylation activity, we purified recombinant full-length MettL16 (Fig. 4*A*) and performed the enzyme assays using the first hairpin of the MTA2A 3’ UTR, as previously used in the structural characterization (48), under three different buffer conditions side by side. The target sequence of MettL16 in the 3’ UTR of MAT2A mRNA is well conserved among the Mammalia, as previously noted (36, 37) (Fig. 4*B*). MettL16 exhibited the highest activity in the absence of divalent metal ions (buffer 3), showed decreased activity at 10 mM MgCl_2_ (buffer 2), and had only barely detectable activity at 50 μM ZnCl_2_ (buffer 1) (Fig. 4*C*). Next, we further investigated the inhibitory effect of zinc ion at two different concentrations (50 μM and 10 mM), together with other divalent metal ions (Mn^2+^, Mg^2+^, and Ca^2+^) (Fig. 4*D*). Zinc had the most deleterious effect, manganese (Mn) yielded significant inhibition at 10 mM, whereas magnesium (Mg) and calcium were tolerated. Nevertheless, no metal ions are required for MettL16 activity. It is not clear if the Zn effect is on the enzyme itself (50) or the RNA substrate—metal ions can have substantial effects on RNA conformation (51). We note that there is no metal binding site(s) identified in the structures of MettL16 bound to either RNA or SAH. Similarly, we find that zinc also inhibits the activities of PCIF1 and MettL5-Trm112 (Figs. 1*H* and 3*H*).

MettL16 has a broad pH optimum range (5.4–9.4), with only slightly higher activity at pH 8.0 (Fig. 4*E*). Unlike MettL5 (optimum <50 mM NaCl) and PCIF1 (optimum <200 mM NaCl), MettL16 has a relatively higher activity over a range of 200 to 500 mM NaCl with a small rise of activity at 400 mM NaCl (Fig. 4*F*). Furthermore, MettL16 may use a random sequential reaction mechanism, in which the binding order of two substrates (SAM and RNA) does not matter (Fig. 4*G*). Next, we tested that the reaction time we used (10–30 min) is within a linear range for varied RNA concentrations (Fig. 4*H*). Importantly, under the optimized laboratory conditions (pH 8.0, 400 mM NaCl and t = 20 min), MettL16 has a nearly linear relationship with the concentration of SAM in the range of concentrations tested (up to 0.4 mM; *i.e.*, *K*_m_ > 0.4 mM for SAM; Fig. 4*I*). This linearity is in contrast with the hyperbolic relationships observed for PCIF1 (Fig. 1*E*) and MettL5 (Fig. 3*E*), both of which have a *K*_m_ value of ∼1 μM for SAM. In addition, MettL16 also has a high *K*_m_ value for RNA (∼10 μM; Fig. 4*J*). Although unusual for a SAM-dependent enzyme-catalyzed methylation reaction, the nearly linear relationship between the rate of reaction and the SAM concentration reflects the specific function of MettL16 in regulating intracellular SAM concentration. Specifically, as noted above, higher SAM concentration leads to higher MettL16 turnover on MAT2A pre-mRNA, reducing the lifetime of the MettL16-RNA complex, in turn resulting in less splicing and more degradation of MAT2A mRNA, and ending in reduced SAM synthetase levels and reduced SAM biosynthesis.

## Discussion

To date, considerable efforts have been made to develop therapeutic agents targeting SAM-dependent MTases, abnormal activities of which underlie the pathology of various diseases, including cancer, diabetes, and Alzheimer’s syndrome (52). High-throughput compound screening is a powerful method to identify initial hits and the inhibitor selectivity against one particular target over a panel of MTases is an important initial step for further optimization. Given the variation in optimal reaction conditions reported here, we suggest that optimizing assay conditions for individual MTases (whether the target enzyme or control enzymes) is an essential step in reducing false-positive or -negative hits (53). Here we demonstrated that three human RNA MTases have both common and unique reaction preferences and kinetic parameters *in vitro*.

To investigate *in vitro* methylation activities of SAM-dependent MTases, we suggest starting with minimal buffer components, considering ionic strength, pH, temperature, and metal ions. In our own practice, we purify and store recombinant proteins in high salt and anything else needed for protein stability. However, the methylation activities of most SAM-dependent MTases we have studied are salt-sensitive. For example, Dim-5, a histone lysine methylase, is extremely sensitive to salt, and 50 to 100 mM NaCl inhibits its activity ∼65% to 95% (54). In this study, 100 mM NaCl inhibited MettL5-Trm112 activity about 80%, and at 200 mM NaCl no activity was observed (Fig. 3*B*). Our Dnmt3a methylation assays were carried out with no added salt (55) or 40 mM NaCl (56). Directly related to this study is our personal experience with *E. coli* EcoGII MTase, which showed no activity in the initial assay, but yielded activity under the condition of 50 mM HEPES pH 7.0 and 1 mM EDTA (57).

The optimum pH may be substrate-specific. Dim-5 shows maximal activity on histone lysine at ∼pH 9.8 (54). SETD3 has optimal pH at ≥ 7 for histidine methylation, but 10.5 for lysine methylation (58). HemK2-Trm112 complex is most active for glutamine methylation at pH 8.0 but for lysine methylation at pH 10.5 (15). In this study, PCIF1 showed higher activity on mRNA cap analog at low (5.4) and high pH (9.4) than at intermediate pH levels. This unusual pattern might be related to the fact that there are two titratable groups on the cap ligand directly involved in the binding (41), which our SAXS and EM models suggest may involve a highly conserved basic face on the carboxy-terminal helix. It is worth noting that pH and ionic strength are not entirely independent variables (59). We would also note that optimal *in vitro* conditions for each enzyme, acting on a specific substrate, likely differ from *in vivo* “physiological” conditions, whether in bacteria or mammalian cells. This can result from parameters ranging from ionic differences (60) to macromolecular crowding (61). The optimal conditions might also imitate the environment within macromolecular complexes, within which many enzymes act *in vivo* (62).

Our observation of a pH minimum for PCIF1 activity, flanked by two (relative) optima, is unusual among MTases, and we ruled out possible pH-dependent aggregation. The reduction in activity from pH 5.4 to 8.0 can be explained by apparently lower substrate binding (Fig. 1*F*). The increased activity between pH 8.0 and 9.4 is not associated with changes in *K*_m_ for either substrate (RNA or SAM) and is seen with both the mRNA cap and oligo RNA substrates. It might reflect increased ease of deprotonating the target adenine amino group (NH_2_) in the environment of the PCIF1 catalytic pocket.

Temperature is another variable to consider if no *in vitro* activity is observed. Dim-5 is most active *in vitro* at ∼10 °C and nearly inactive at 37 °C (54). In this study, we used room temperature (∼22 °C) for convenience, even though the three enzymes studied here have higher activity at 37 °C (Figs. 1*I*, 3*I* and 4*K*).

Divalent metal ions are not commonly required for SAM-dependent methylation and in particular are not known to be important for N-methylation. In this study, we showed that Zn^2+^ is strongly inhibitory toward all three enzymes, while Mn^2+^ at 10 mM inhibited MettL16 activity about 65%, and Mg^2+^ and Ca^2+^ had minimal (negative) effects (Fig. 4*C*). However, metal ions *are* involved in some (small molecule) O-methylations. For example, an Mg^2+^ ion bound to catechol O-MTase is required for substrate binding and/or reaction (63), and magnesium is required for O-linked methylation of sugar substituents (64, 65). A calcium ion is observed in the active site of caffeoyl coenzyme-A 3-O-MTase; the activity of which can be observed in the presence of Mg^2+^, Ca^2+^, or Zn^2+^, but that activity is significantly reduced by Mn^2+^ (66). In contrast, metal-independent O-methylation has also been found in the rebeccamycin sugar 4′-O-MTase RebM (67) and mRNA cap-specific ribose 2’-O-MTase VP39 (68).

In summary, the three human RNA N6mA MTases studied here illustrate the wide range of optima for various reaction conditions, which has implications for their study and for inhibitor screens.

## Experimental procedures

### Expression and purification

Human PCIF1 (NP_071387.1 or BAC45238.1) was cloned into pGEX-6p1 vector with an N-terminal GST tag (pXC2055). Human MettL5 (NP_001280115.1) was cloned into pET28a vector with a C-terminal His_6_ tag (pXC2062), and human Trm112 (NP_057488.1) was cloned in pET22b vector without any fused tag (pXC2076), then two plasmids were cotransformed into *E. coli* cells. Human MettL16 (NP_076991.3) was cloned into the pET22b vector with a C-terminal His_6_-tag (pXC2210).

All plasmids used in this study were transformed into *E. coli* BL21(DE3) CodonPlusTM cells (Stratagene). Expressions of fusion proteins (either GST-PCIF1 or MettL5-His_6_ and MettL16-His_6_) were performed at 16 °C overnight after induction with 0.1 mM isopropyl β-D-1-thiogalactopyranoside. All purification steps were performed at 4 °C and conducted in a BIO-RAD NGC system using a three-column chromatography of affinity, ion exchange, and size exclusion.

For purification of PCIF1, the cell culture was harvested and sonicated in a lysis buffer of 20 mM Tris-HCl pH 8.0, 150 mM NaCl, 5% glycerol, and 0.5 mM Tris(2-carboxyethyl)phosphine (TCEP). The centrifugation supernatant was loaded onto a self-packed glutathione-Sepharose column (Cytiva), and GST-PCIF1 was eluted with buffer containing 20 mM reduced glutathione. After removing the GST tag by adding 100 μg PreScission Protease overnight at 4 °C, leaving five additional N-terminal residues (Gly-Pro-Leu-Gly-Ser), the cleaved protein solution was diluted to 50 mM NaCl, loaded onto a HiTrap SP-HP column (5-ml volume, GE Healthcare), and then PCIF1 protein was eluted in an NaCl gradient at 150 mM. PCIF1 was further purified by Superdex 200 (GE Healthcare) in the lysis buffer (Fig. S2, *A*–*C*). The purified PCIF1 proteins at 6.5 mg/ml (∼80 μM with extinction coefficient of 1.23) were aliquoted and kept at −80 °C for further use.

Purification steps for Mettl15-Trm112 and Mettl16 were similar. The cells were pelleted and sonicated in the lysis buffer described above but containing 150 mM NaCl and 50 mM imidazole (MettL5-Trm112) or the lysis buffer containing 500 mM NaCl and 20 mM imidazole (MettL16). After centrifugation, the supernatant was loaded onto a Ni-NTA affinity column (QIAGEN), washed, and then eluted with either 250 mM imidazole (MettL5-Trm112) or 100 mM imidazole (MettL16). Then, the solution containing the protein complex or the C-terminal His_6_-tagged protein was diluted to 100 mM NaCl and loaded onto a HiTrap Q-HP column (5-ml column volume, GE Healthcare). The MettL5-Trm112 complex was eluted from the HiTrap Q-HP in an NaCl gradient at 300 mM, subsequently purified by Superdex 200 (GE Healthcare) in the lysis buffer at 150 mM NaCl, and then concentrated to 1.77 mg/ml (∼40 μM with extinction coefficient of 0.52) for storage. Mettl16-His_6_ protein was eluted in an NaCl gradient at 200 mM, further purified by Superdex 200 (GE Healthcare) in lysis buffer with 200 mM NaCl, and concentrated to 1.3 mg/ml (∼20 μM with extinction coefficient 0.99).

### SAM-dependent methylation assays

The Promega bioluminescence assay (MTase-Glo) (69) was used to measure the methylation reaction by-product SAH, which is converted into ATP in a two-step reaction, and ATP is then detected by a luciferase reaction. A low-volume 384-well plate with each well containing an aliquot of 5 μl of reaction mixture was used to measure luminescence signal by a Synergy 4 multimode microplate reader (BioTek). The MTase-Glo assay produced lower false-positive rates compared with other methods (70) and the substrate-independent assay has been used in our previous studies on peptide methylation (58, 71, 72) and DNA methylation (56). All methylation reactions were terminated by addition of trifluoroacetic acid (TFA) to a final concentration of 0.1% (v/v).

The oligonucleotides used in assays were synthesized by Integrated DNA Technologies, Inc. The mRNA cap analog was purchased from TriLink BioTechnologies (catalog number N-7113).

### Methylation reactions of PCIF1

A 20 μl reaction mixture contained 0.2 μM PCIF1, 4 μM mRNA cap analog, 20 μM SAM, 50 mM NaCl, and 1 mM DTT and was adjusted to varied pH in three buffer systems: (1) 20 mM Tris-HCl (below pH 9.4), (2) 20 mM citric acid, bis-tris propane (below pH 9.4), or (3) 20 mM glycine/NaOH (above pH 9). The 20 mM citric acid, bis-tris propane is a mixture of 10 mM citric acid and 10 mM Bis-Tris propane. The reaction was incubated at room temperature (∼22 °C) for 20 min. To test the effect of ionic strength, reactions were carried out in 20 mM Tris-HCl pH 8.0 with NaCl concentrations varying from 50 mM to 1.0 M.

Steady-state kinetic reactions were carried out in 20 μl mixtures at three pH conditions (5.4, 8.0, or 9.4), in 20 mM citric acid and 20 mM Bis-Tris propane, 1 mM DTT, 50 mM NaCl, 50 nM PCIF1, and either 20 μM SAM with varying mRNA cap concentrations or 10 μM RNA with varying SAM concentrations. The reactions proceeded at room temperature for 10 min. The dependence of the product formation per enzyme on substrate concentration was analyzed according to Michaelis–Menten kinetics using GraphPad Prism 8.

Reactions with RNA oligonucleotide without cap modification were carried out in a mixture containing 20 mM Tris-HCl, pH 8.0, 1 mM DTT, 50 mM NaCl, 2 μM PCIF1, 20 μM SAM, and 10 μM RNA, at room temperature for 4 h. An oligo that did not contain adenosine was used as negative control. The luminescence value of the reaction in the absence of oligo substrate was taken as the background and subtracted from the measured luminescence values of the reactions in the presence of oligo substrate.

### Methylation reactions of MettL5-Trm112

Single-stranded RNA, corresponding to the sequence surrounding A_1832_ of 18S rRNA (5’-UCGUA**A**CAAGGUUU-3’), was used as a substrate for MettL5-Trm112 complex. Reactions were carried out in 20 μl mixtures containing 20 mM Tris-HCl, pH 8.0, 1 mM DTT, and 0.2 μM enzyme complex, using either 40 μM SAM with varying RNA concentration or 10 μM RNA with varying SAM concentration. The reactions proceeded at room temperature for 20 min.

### Methylation reactions of MettL16

The reaction assays with MettL16 were carried out in a 20-μL reaction mixture containing 20 mM Tris-HCl pH 8.0 and 1 mM DTT at room temperature, under low or high ionic strengths (50 mM or 400 mM NaCl), varied concentrations of SAM (20 μM, 100 μM or 1 mM) and of the hairpin RNA substrate (5, 10 or 20 μM), and varied reaction times (20 min, 2 h or 4 h).

### Size-exclusion chromatography coupled small-angle X-ray scattering and multiangle light scattering (SEC-SAXS-MALS)

Purified PCIF1 at 4 mg/ml (60 μl) in 20 mM Tris-HCl, pH 7.5, 150 mM NaCl, and 0.1% β-mercaptoethanol was loaded onto a Shodex KW803 column of Agilent 1260 Infinity HPLC system coupled to the SAXS flow cell. The column was equilibrated with the same buffer at a flow rate of 0.4 ml/min. A total of 55 μl of sample was analyzed. The SAXS scattering data were collected at the ALS Beamline 12.3.1 at 20 °C (73). X-ray wavelength was set at 1.127 Å and the sample to detector distance was set at 2100 mm, resulting in scattering vectors, q, ranging from 0.01 Å^−1^ to 0.4 Å^−1^. The 3 s X-ray exposures were collected continuously during a duration of 30 min elution. ScÅtter (http://www.bioisis.net/) was used to subtract the background scattering from frames recorded prior to the protein elution peak.

The SAXS data were analyzed using the programs available in the ATSAS 3.0.2 suite (74). PRIMUS calculated the radius of gyration (Rg) on the basis of Guinier approximation. GNOM calculated the distance distribution function (Dmax). DATMOW calculated the molecular mass. DAMMIF (75) built 20 independent *ab initio* models, which were further averaged and filtered by DAMAVER (76) and refined by DAMMIN (77). PyMol (Schrödinger, LLC) and UCSF Chimera (78) were used for structural visualization, analysis, and figure preparation.

For MALS experiments, an 18-angle DAWN HELEOS II light scattering detector was connected in tandem to an Optilab refractive index concentration detector (Wyatt Technology). System normalization and calibration were performed with bovine serum albumin at 10 mg/ml (45 μl) in the same running buffer. The UV, MALS, and differential refractive index data were analyzed using Wyatt Astra 7 software to monitor the homogeneity of the sample across the elution peak and calculate the molar mass of the PCIF1.

### Negative stain transmission electron microscopy

Purified PCIF1 at 0.05 mg/ml (3 μl) was adsorbed onto the glow discharged carbon-coated grid (CF200-CU, Electron Microscopy Sciences) for 60 s and then blotted with Whatman filter paper 541 (Millipore Sigma). The grids were washed with two drops of water, with intermittent blotting, followed by staining with three drops of 2% uranyl acetate, and then blotted to remove the excess stain and air-dried in the fume hood. The negatively stained grids were imaged at room temperature using a JEM 2100 transmission electron microscope operating at 200 keV (JOEL) and equipped with a LaB6 filament, 3k x 4k Direct Electron Detector (DE12), and a Gatan 4k x 4k charged-coupled device (CCD). Images were recorded at a magnification of 40,000×. All the steps of image processing were carried out using cryoSPARC (79) including estimation of contrast transfer function (CTF), particle picking with the help of blobpicker, particle extraction, 2D classification, *ab initio* 3D reconstruction, and homogeneous refinement. A total of 12,915 particles were classified in ten distinct classes. Among them, a total of 10,976 particles were eventually used to generate the 3D EM map, which was further visualized using UCSF Chimera (78).

### Homology modeling of full-length PCIF1 using I-TASSER

To generate a homology model of the full-length PCIF1, we used the online webserver I-TASSER (44), which uses the multiple threading alignments approach followed by iterative simulations and refinement. I-TASSER generated the top ten threading templates along with their alignments and Z-scores, which were further used to generate the homology model. As expected, the PDB 6IRV of human PCIF1 MTase domain (23) had the best Z-score and was used for further modeling. I-TASSER predicted the top five models with global and local accuracy estimates. The model with the best confidence C-score (0.32) and estimated TM-score (0.8 ± 0.1) was used for manual docking.

### Dynamic light scattering

A DynaPro PlateReader-II (Wyatt Technology Corporation) was used for measurement at 25 °C using the automatic mode. An aliquot of 30 μl of 1 mg/ml PCIF1 buffered at different pH values was add to a 384-well microwell plate. The hydrodynamic radius, molar mass, and polydispersity were calculated using the DYNAMICS software (Wyatt Technology Corporation).

## Data availability

All experimental data are contained within the article.

## Conflict of interest

The authors declare no competing interests with the contents of this article.
