# Supplementary material for: Enzymatic characterization of three human RNA adenosine methyltransferases reveals diverse substrate affinities and reaction optima
Source: J Biol Chem. 2021 Jan 9;296:100270. doi: 10.1016/j.jbc.2021.100270 (PMC7948815; doi:10.1016/j.jbc.2021.100270)
Supplement: Supplemental Figures S1–S4 [file mmc1.pdf]

Supporting Information

Enzymatic characterization of three human RNA adenosine methyltransferases reveals diverse substrate affinities and reaction optima

Dan Yu[1], Gundeep Kaur[1], Robert M. Blumenthal[2], Xing Zhang[1,*], Xiaodong Cheng[1,*]

[1]Department of Epigenetics and Molecular Carcinogenesis, University of Texas MD Anderson Cancer Center, Houston, TX 77030, USA

[2]Department of Medical Microbiology and Immunology, and Program in Bioinformatics, The University of Toledo College of Medicine and Life Sciences, Toledo, OH 43614, USA

* Correspondence: XZhang21@mdanderson.org or XCheng5@mdanderson.org

Email addresses:

DY (DYu6@mdanderson.org); GK (GKaur1@mdanderson.org);

RMB (Robert.Blumenthal@utoledo.edu); XZ (XZhang21@mdanderson.org);

XC (XCheng5@mdanderson.org)

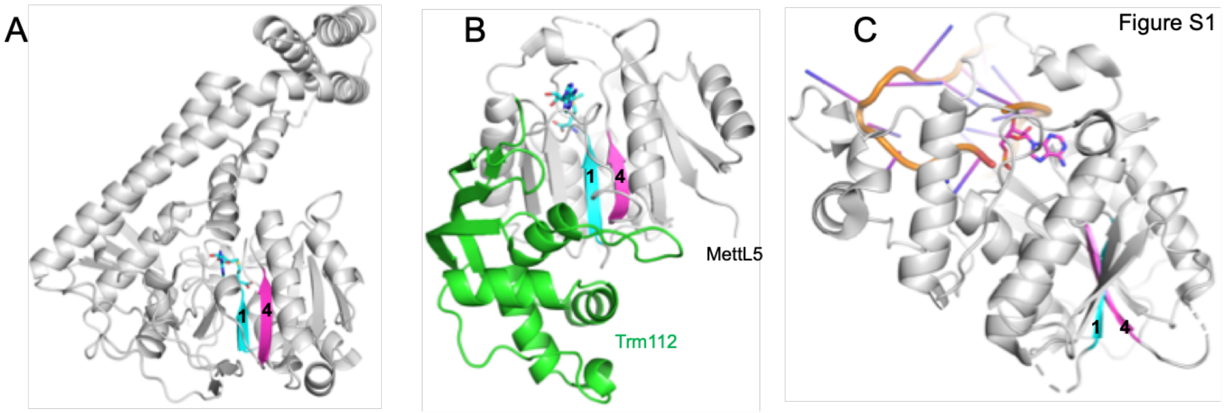

Human PCIF1 (PDB 6IRW)
(mRNA cap N6mA)

Human MettL5-Trm112 (PDB 6H2U)
(18S rRNA N6mA)

Human MettL16 (PDB 6DU4)
(3' UTR hairpin N6mA)

D Group α or γ MTases = NH_2 • • • • β1-2-3 • • • • • • • • • • • • • β4-5-6-7 • • •COOH

```
                   β1 Motif I                   β4 Motif IV          substrate/product
E. Coli Dam   31 LVEPFVGAGSVFLNT 45.......175 SSVVYCDPPYAPLSAT 190  DNA       N6mA
MettL5        53 KVVADLGCGCGVLSI 67.......120 FDTVIMNPPFGTKNNK 135  rRNA      N6mA
MettL16      104 RRGIDIGTGASCIYP 118......178 YDFCMCNPPFFANQLE 193  snRNA     N6mA
HemK2         47 EICLEVGSGSGVVSA 61.......116 VDLLVFNPPYVVTPPQ 131  protein   Q/K(me1)
PCIF1/CAPAM  513 TFECFASPLNCYFKQ 527......547 SGSFEANPPFCEELMD 562  mRNA cap  N6mA(m)
```

E

Mammal	PCIF1	Motif I	Motif IV
Platypus – *Ornithorhynchus anatinus*	XP_028904763	HRLFGVSFEC**FASPL**NCYFRQYCSA	FSPLSGSFEA**NPPF**CEELMDAMVS
Opossum – *Monodelphis domestica*	XP_007474986	HRLFGVSFEC**FASPL**NCYFRQYCSA	FYPLSGSFEA**NPPF**CEELMDAMVS
Elephant – *Loxodonta africana*	XP_023406075	HRLFGVSFEC**FASPL**NCYFRQYCSA	FSPLSGSFEA**NPPF**CEELMDATVS
Armadillo – *Dasypus novemcinctus*	XP_012385012	HRLFSVSFEC**FASPL**NCYFRQYCSA	FSPLSGSFEA**NPPF**CEELMDAMVS
**Human – *Homo sapiens***	NP_071387	HRLFGVSFEC**FASPL**NCYFRQYCSA	FAPLSGSFEA**NPPF**CEELMDAMVS
Mouse – *Mus musculus*	NP_001361055	HRLFGVSFEC**FASPL**NCYFRQYCSA	FTPLSGSFEA**NPPF**CEELMDAMVS
Mole – *Condylura cristata*	XP_004687490	HRLFGVSFEC**FASPL**NCYFRQYCSA	FSPLSGSFEA**NPPF**CEELMDAMVS
Bat – *Pteropus vampyrus*	XP_011367609	HRLFGVSFEC**FASPL**NCYFRQYCSA	FSPLSGSFEA**NPPF**CEELMDAMVS
Goat – *Capra aegagrus / C. hircus*	XP_017913438	HRLFGVSFEC**FASPL**NCYFRQYCSA	FSPLSGSFEA**NPPF**CEELMDAMVS
Horse – *Equus caballus*	XP_023482399	HRLFGVSFEC**FASPL**NCYFRQYCSA	FSPLSGSFEA**NPPF**CEELMDAMVS
Pangolin – *Manis pentadactyla*	XP_036758026	HRLFSVSFEC**FASPL**NCYFRQYCSA	FSPLSGSFEA**NPPF**CEELMDAMVS
Wolf – *Canis lupus*	XP_005635184	HRLFSVSFEC**FASPL**NCYFRQYCSA	FSPLSGSFEA**NPPF**CEELMDAMVS

Mammal	MettL5	Motif I	Motif IV
Platypus – *Ornithorhynchus anatinus*	XP_028927764	YDDIEDKVVA**DLGCG**CGVLSIGSAM	MSKSFDTVIM**NPPF**GTKHNKGMDM
Opossum – *Monodelphis domestica*	XP_001375717	YDDIENKVIG**DLGCG**CGMLSIGAAM	ISKSFDTIIM**NPPF**GTKHNKGMDM
Elephant – *Loxodonta africana*	XP_010601144	YDDIENKVVA**DLGCG**CGVLSIGTAM	MSKSFDTVIM**NPPF**GTKNNKGTDM
Armadillo – *Dasypus novemcinctus*	XP_004460848	YDDIENKVVA**DLGCG**CGVLSIGTAM	MSKSFDTVIM**NPPF**GTKNNKGIDI
**Human – *Homo sapiens***	NP_001280115	YDDIENKVVA**DLGCG**CGVLSIGTAM	MSKSFDTVIM**NPPF**GTKNNKGTDM
Mouse – *Mus musculus*	NP_083556	YDDIENKAVA**DLGCG**CGVLSIGAAM	MSKLFDTVIM**NPPF**GTKNNKGTDM
Mole – *Condylura cristata*	XP_004674616	YDDIENKMVA**DLGCG**CGVLSIGTAM	ISKSFDTVIM**NPPF**GTKNNKGTDM
Bat – *Pteropus vampyrus*	XP_023385363	YDDIENKVIA**DLGCG**CGVLSIGTAM	MSKSFDTVIM**NPPF**GTKNNKGTDM
Goat – *Capra aegagrus / C. hircus*	XP_005676044	YDDIENKVVA**DLGCG**CGVLSIGTAM	LSKSFDTVIM**NPPF**GTKNNKGTDM
Horse – *Equus caballus*	XP_001498013	YDDIENKVVA**DLGCG**CGVLSIGTAM	MSKSFDTVIM**NPPF**GTKNNKGTDM
Pangolin – *Manis pentadactyla*	XP_036743976	YDDIENKVVA**DLGCG**CGVLSIGTAM	MSKSFDTVIM**NPPF**GTKN-KGTDM
Wolf – *Canis lupus*	XP_005640349	YDDIENKVVA**DLGCG**CGVLSIGTAM	MSKSFDTVIM**NPPF**GTKNNKGTDM

Mammal	MettL16	Motif I	Motif IV
Platypus – *Ornithorhynchus anatinus*	XP_028938135	SLKNTLRRGI**DIGTG**ASCIYPLLGA	SEIIYDFCMC**NPPF**FANQLEAKGV
Opossum – *Monodelphis domestica*	XP_016284792	PARSALRRGI**DIGTG**ASCIYPLLGA	SEIIYDFCMC**NPPF**FANQLEAKGV
Elephant – *Loxodonta africana*	XP_003417034	SDRSTLRRGI**DIGTG**ASCIYPLLGA	SEIIYDFCMC**NPPF**FANQLEAKGV
**Human – *Homo sapiens***	NP_076991	SDKSTLRRGI**DIGTG**ASCIYPLLGA	SEIIYDFCMC**NPPF**FANQLEAKGV
Mouse – *Mus musculus*	NP_080473	SDKTTLRRGI**DIGTG**ASCIYPLLGA	SEIVYDFCMC**NPPF**FANQLEAKGV
Mole – *Condylura cristata*	XP_004684839	SDKSTLRRGI**DIGTG**ASCIYPLLGT	SEIIYDFCMC**NPPF**FANQLEAKGV
Bat – *Pteropus vampyrus*	XP_011358139	SDKTTLRRGI**DIGTG**ASCIYPLLGS	SEIVYDFCMC**NPPF**FANQLEAKGV
Goat – *Capra aegagrus / C. hircus*	XP_005693382	SDKSTLRRGI**DIGTG**ASCIYPLLGT	SEIIYDFCMC**NPPF**FANQLEAKGV
Horse – *Equus caballus*	XP_014583405	SDKSTLRRGI**DIGTG**ASCIYPLLGT	SEIIYDFCMC**NPPF**FANQLEAKGV
Pangolin – *Manis pentadactyla*	XP_036762024	SDKSTLRRGI**DIGTG**ASCIYPLLGT	SEIIYDFCMC**NPPF**FANQLEAKGV
Wolf – *Canis lupus*	XP_022279183	SDKSTLRRGI**DIGTG**ASCIYPLLGT	SEIIYDFCMC**NPPF**FANQLEAKGV

Figure S1. The seven-stranded class-I methyltransferase domain in (**A**) PCIF1 (PDB 6IRW), (**B**) MettL5-Trm112 (PDB 6H2U), and (**C**) Mettl16 (6DU4), showing the standard central

topological switch-point, seen in this family of MTases, between strands β1 (cyan) and β4 (magenta). (**D**) The relative sequence positions, of conserved motif I (located in the carboxy end of strand β1) and motif IV (located in carboxy end of strand β4), are similar to those of *E. coli* DNA adenine methyltransferase (Dam) and human HemK2, a protein glutamine and lysine MTase. The common feature of the respective substrates is the amino group (NH_2) of DNA/RNA adenine or protein glutamine or lysine (in the deprotonated state), at which all three methylation reactions are catalyzed by a NPPF/Y version of motif IV. (**E**) Structure-guided alignment shows that PCIF1 has a somewhat unusual version of motif I (SAM binding, in this case lacking the typical GxG element) that is highly conserved among mammals. The MettL5 and MettL16 motifs I (DxGxG) and IV are well conserved across the Mammalia. Not shown is *D. novemcinctus* (armadillo) MettL16 homolog (XP_023441943), which may start from the wrong (downstream) ATG; the rest of the putative amino-terminal sequence is not available. Variations from the human sequence are highlighted by cyan background.

Figure S2

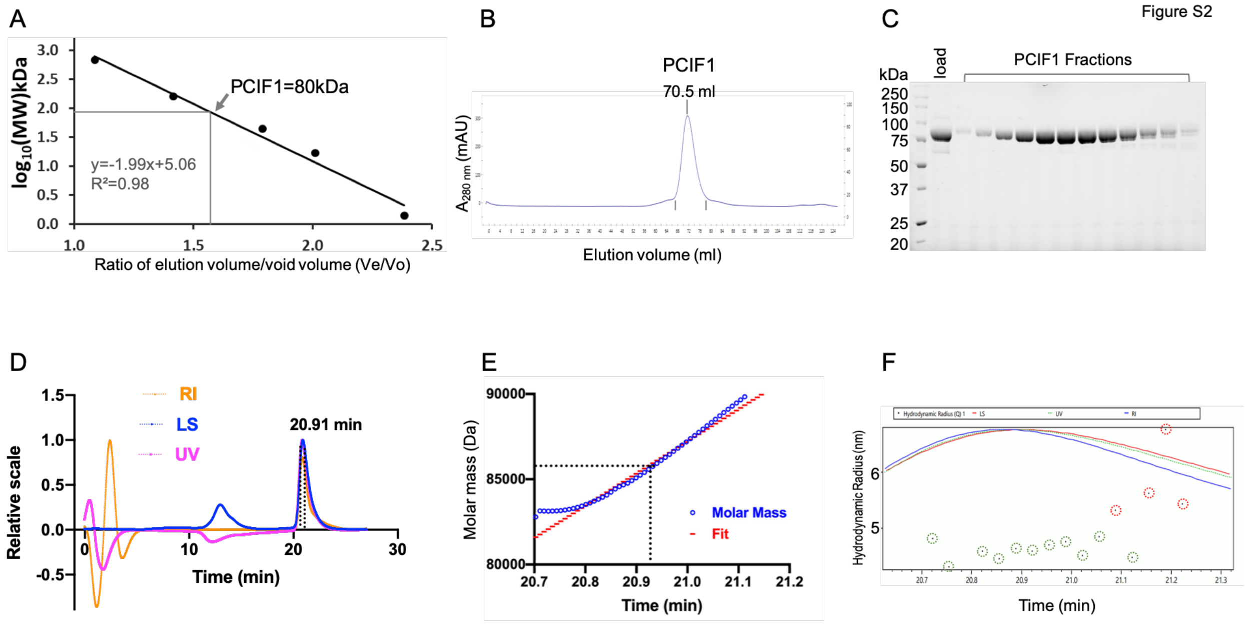

Figure S2. Purified recombinant PCIF1 used in this study. (**A**) A calibration curve for a Superdex 200 16/60 column based on five standards. The estimated apparent molecular weight of PCIF1 is 80 kDa. (**B**) Elution profile of PCIF1 and (**C**) peak fractions (shown in 12% SDS PAGE) from gel filtration column (Superdex S200 16/60). **(D)** SEC-MALS profile of PCIF1. Elution profile from the Shodex KW803 column. Peak at 20.91 min corresponding to PCIF1. (**E**) The observed molecular weight of PCIF1 is 86 kDa (Y-axis) at 20.91 min (X-axis). (**F**) Plot of hydrodynamic radius in circles (Y-axis) versus time of elution (X-axis). The observed hydrodynamic radius is 4.8±0.4 nm.

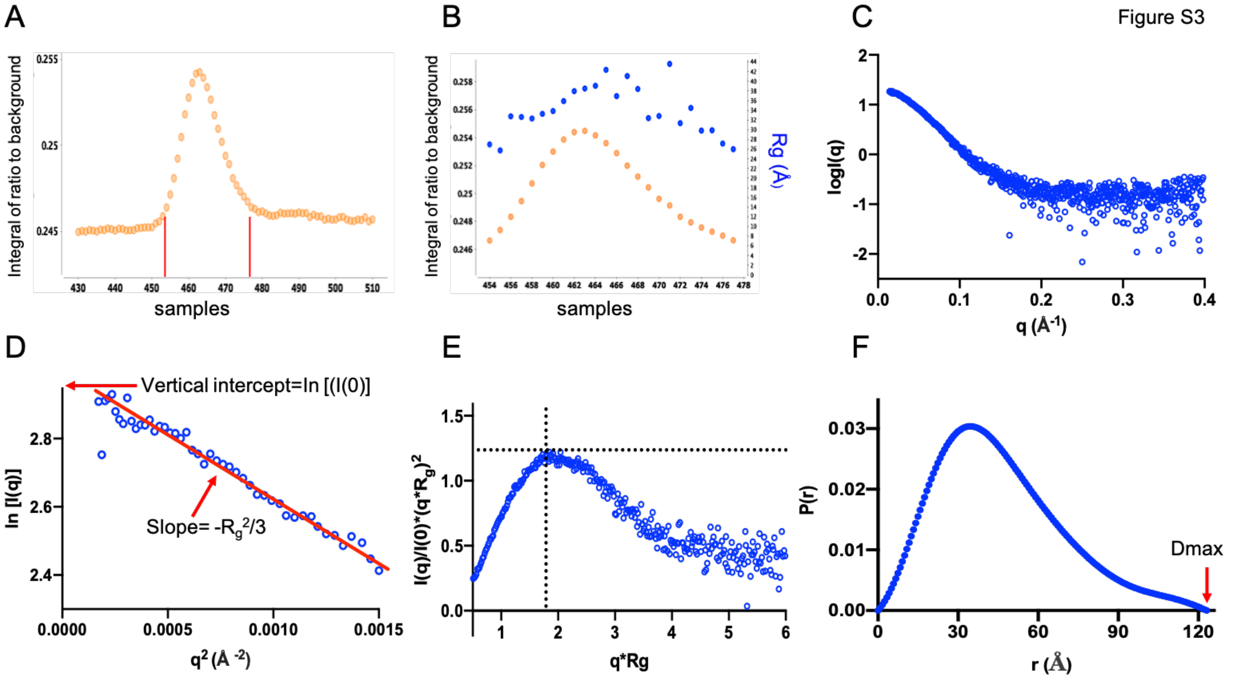

Figure S3. SEC-SAXS analysis of PCIF1. (**A**) In line SEC trace showing PCIF1 elutes as a single peak with no apparent signs of aggregation. (**B**) The blue dots show corresponding values of radius of gyration (R_g) across the elution peak. (**C**) Experimental scattering curve plotted as logI(q) versus q in units of inverse angstroms after background-subtracted from the buffer scattering ($q=4\pi\sin\theta/\lambda$ where 2θ is defined as the scattering angle and λ is the wavelength in Å of the incoming X-ray beam). (**D**) Guinier plot as a linear fit between In(I) versus q^2. The R_g value (~36 Å) is derived from the slope (= $-R_g^2/3$). The zero-angle scattering intensity I(0) value (20.81) is derived from the vertical intercept. (**E**) Dimensionless Kratky plot is presented as $(qR_g)^2I(q)/I(0)$ versus qR_g. The bell-shaped peak at low q suggests that PCIF1 is well-folded and globular in solution. The Porod volume is ~125,875 $Å^3$ and molecular weight is ~86 kDa. (**F**) Normalized pair distance distribution function P(r), generated from the Fourier transform of I(q), represents a continuous electron-pair distances (r) in the protein. The maximum dimension of the protein Dmax is estimated to be 121 Å.

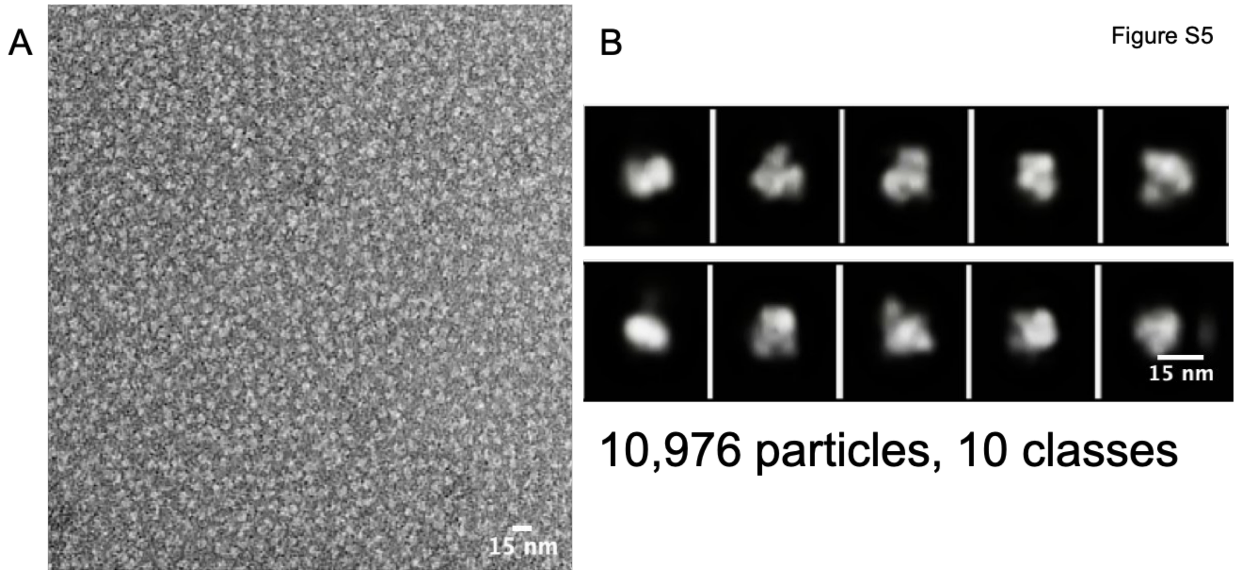

Figure S4. EM analysis of full length PCIF1. (**A**) A representative electron micrograph of PCIF1 negatively stained with 2% uranyl acetate and recorded at 40,000x. The scale bar is 15 nm. (**B**) Ten different 2D class averages generated from 10,976 particles.
